# P-298. Integrating HIV Pre-exposure Prophylaxis into Graduate Medical Resident Education: Is It Prime Time?

**DOI:** 10.1093/ofid/ofaf695.518

**Published:** 2026-01-11

**Authors:** Diane Choi, Lillian Seo, Amanda Westlake, Armando Paez

**Affiliations:** Baystate Medical Center, Northampton, MA; Baystate Medical Center, Northampton, MA; UMass Chan Baystate Medical Center, Springfield, Massachusetts; UMass Chan Medical School - Baystate, Springfield, Massachusetts

## Abstract

**Background:**

Pre-exposure prophylaxis (PrEP) has been a significant advancement in the prevention of HIV infection. However, knowledge and attitudes among physicians regarding PrEP options and lack of PrEP education in residency curricula may limit adoption of this preventive service. The primary aim of this project is to assess the impact of asynchronous education of internal medicine (IM) and medicine-pediatrics (Med-Peds) residents on HIV PrEP prescribing practices.

**Figure 1.**
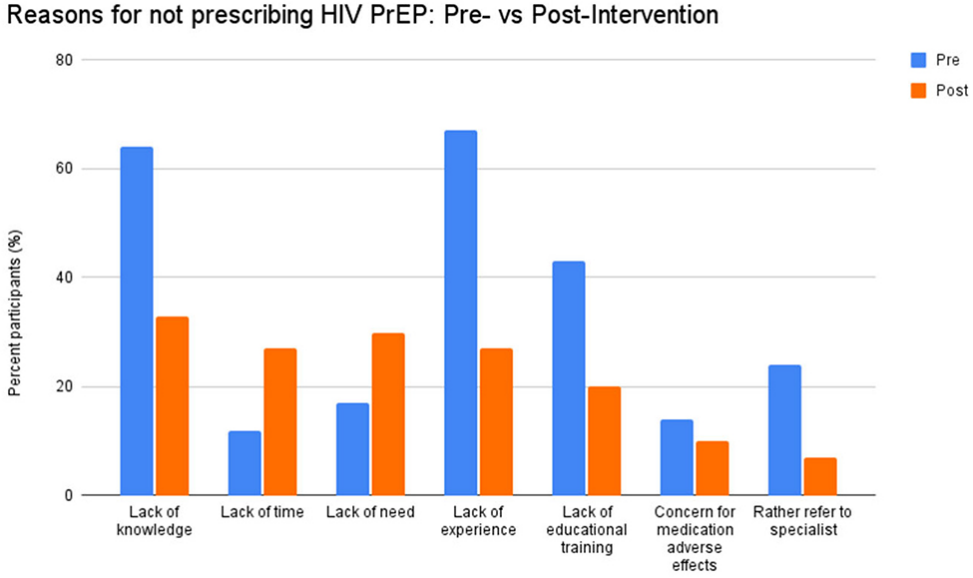
Differences in perceived barriers to not prescribing HIV PrEP for pre- (blue) vs post-intervention (orange) participants

**Figure 2.**
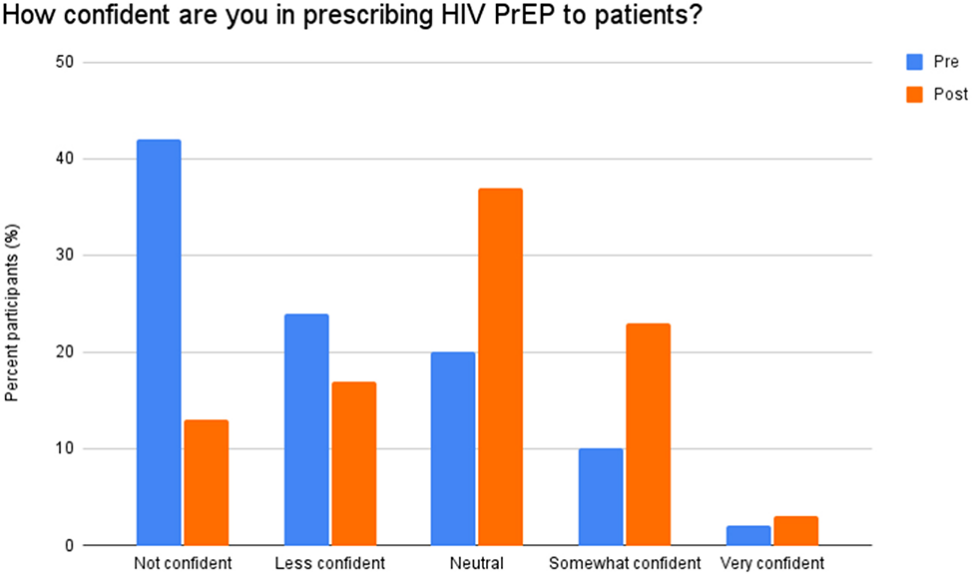
Differences in confidence levels in prescribing HIV PrEP for pre- (blue) vs post-intervention (orange) participants

**Methods:**

Asynchronous education consisted of an in-person lecture, an online case-based module, and dissemination of a standardized clinical practice guideline to IM and Med-Peds residents and teaching attendings at three community health clinics within Baystate Health system. The impact was assessed through cross-sectional, anonymous online surveys via Research Electronic Data Capture, evaluating knowledge, attitudes, and beliefs about HIV PrEP before and four months after the educational campaign.

**Figure 3.**
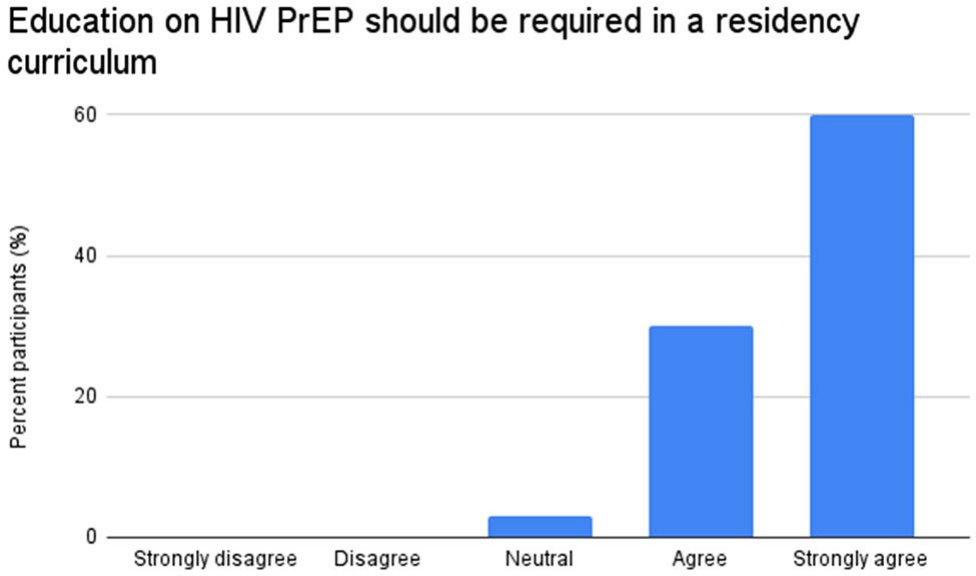
Attitudes on incorporating HIV PrEP education into residency curriculum among post-intervention participants

**Results:**

There were 42 and 30 participants in pre-intervention and post-intervention surveys, respectively. 60% of post-intervention respondents completed at least one component of the educational campaign. Significant barriers to prescribing identified in the pre-intervention survey included lack of knowledge (64%) and lack of education (43%). In comparison, after the intervention, 33% and 20% of participants cited lack of knowledge and lack of education, respectively (Figure 1). 66% of residents reported they were “Not confident” or “Less confident” in their prescribing abilities before the intervention. After the intervention, only 30% were “Not confident” or “Less confident” (Figure 2). Lastly, 90% of participants indicated that they "Agreed" or "Strongly Agreed" that PrEP education should be a required component of the residency curriculum (Figure 3).

**Conclusion:**

Understanding baseline knowledge and attitudes toward prescribing HIV PrEP and perceived barriers in the trainee setting has important implications for community health and graduate medical education. Our project highlights the critical need to incorporate HIV PrEP education into residency training. Key limitations included incomplete delivery of the educational intervention to all intended participants and a small sample size.

**Disclosures:**

Armando Paez, MD, AstraZeneca: Grant/Research Support|Nanobiosym: Grant/Research Support

